# Understanding the benefits of extrinsic emotion regulation in depression

**DOI:** 10.3389/fpsyg.2023.1120653

**Published:** 2023-04-27

**Authors:** Atheer Massarwe, Noga Cohen

**Affiliations:** ^1^Department of Special Education, Faculty of Education, University of Haifa, Haifa, Israel; ^2^The Edmond J. Safra Brain Research Center for the Study of Learning Disabilities, University of Haifa, Haifa, Israel

**Keywords:** extrinsic emotion regulation, depression, intrinsic emotion regulation, reward, empathy, neuroimaging

## Abstract

Depression is a serious psychiatric illness that negatively affects people’s feelings, thoughts, and actions. Providing emotion regulation support to others, also termed Extrinsic Emotion Regulation (EER), reduces depressive symptoms such as perseverative thinking and negative mood. In this conceptual review paper, we argue that EER may be especially beneficial for individuals with depression because it enhances the cognitive and affective processes known to be impaired in depression. Behavioral studies have shown that EER recruits processes related to cognitive empathy, intrinsic emotion regulation (IER), and reward, all impaired in depression. Neuroimaging data support these findings by showing that EER recruits brain regions related to these three processes, such as the ventrolateral prefrontal cortex which is associated with IER, the ventral striatum, which is associated with reward-related processes, and medial frontal regions related to cognitive empathy. This conceptual review paper sheds light on the mechanisms underlying the effectiveness of EER for individuals with depression and therefore offers novel avenues for treatment.

## 1. Introduction

Major Depressive Disorder (MDD) is a highly prevalent and often chronic disorder affecting more than 264 million people worldwide (Santomauro et al., 2021). Depression is therefore considered one of the most pressing health issues of our time (Fried et al., 2022). The increasing rates of depression impose substantial personal and societal costs. Unfortunately, there has been no significant change in the effectiveness of treatment for depression in the last few decades (Khan and Brown, 2015). Approximately half the patients remain depressed after undergoing psychological or pharmacological treatment (Khan and Brown, 2015; Cuijpers et al., 2018). Current treatments reduce the disease burden by only one-third (van Zoonen et al., 2014). In spite of considerable efforts and investment into understanding the biological underpinnings of depression, the mechanisms underlying it remain largely opaque (Kapur et al., 2012; Rogers, 2017). Identifying risk factors and the underlying mechanisms behind depression may assist in the development of effective prevention and treatment approaches (Joormann and Stanton, 2016).

A new approach suggests that individuals with depression can benefit from providing emotion regulation support to others, a process termed interpersonal emotion regulation (Zaki and Craig Williams, 2013) or extrinsic emotion regulation (EER; Nozaki and Mikolajczak, 2020). Based on a comprehensive literature review, we present evidence that EER may be especially helpful for individuals with depression as it enhances three main processes found impaired in depression: cognitive empathy, intrinsic emotion regulation, and reward (for reviews, see Singh and Gotlib, 2014; Cohen and Arbel, 2020).

### 1.1. Extrinsic emotion regulation

Emotion regulation (ER) is the set of processes responsible for monitoring, evaluating, and modifying emotional reactions in order to accomplish one’s goals (Gross and Thompson, 2007). It has recently been proposed that emotional health does not depend on the use of specific emotion regulation strategies but rather on the fit between the strategy used and the situation at hand (Cheng, 2001; Kashdan and Rottenberg, 2010; Bonanno and Burton, 2013; Cheng et al., 2014; Aldao et al., 2015). While most emotion regulation research has focused on intrinsic (or intrapersonal) emotion regulation, in which a person regulates their own emotions, recent studies have started to explore processes related to emotion regulation provided to others during social interactions. This social emotion regulation process, named Extrinsic Emotion Regulation (EER), is defined as “*an action performed with the goal of influencing another person’s emotion trajectory; it can aim to decrease or increase either negative emotion or positive emotion.”* (Nozaki and Mikolajczak, 2020).

EER has been largely studied as a way of reducing negative emotions and increasing positive ones (pro-hedonic motives; Zaki and Craig Williams, 2013). Nevertheless, a provider could also increase the negative emotions of the recipient or decrease the positive ones (contra-hedonic motives; Nozaki and Mikolajczak, 2020). In this manuscript, we discuss only pro-hedonic EER, which is the act of trying to make others feel better.

Relying on the process model of emotion (Gross, 1998), which presents four stages of emotion regulation, EER was also offered to consist of similar stages (Reeck et al., 2016). Namely, EER can be seen as a process consisting of the following stages; identification, selection, implementation and monitoring. In the identification stage, the emotion regulation support provider should infer the recipient’s emotional state, assign a value to that state and establish a regulation goal (Nozaki and Mikolajczak, 2020). In the selection stage, the provider should first perceive the available emotion regulation strategies, evaluate which ones are most valuable, and then choose which ones to use (Tanna and MacCann, 2022). In the implementation stage, the provider should consider how to implement the selected emotion regulation strategies into behavioral tactics, assess their capabilities, limitations and likely outcomes, as well as implement these tactics (Tanna and MacCann, 2022). The last stage is monitoring, in which the provider should identify whether to switch or stop the regulation strategy (Sheppes et al., 2015).

Providing help to others is associated with various beneficial outcomes such as lower mortality (Brown et al., 2003), better overall health (Piferi and Lawler, 2006), improved mood (Schacter and Margolin, 2019), reduced perceived stress (Raposa et al., 2016), greater happiness (Dunn et al., 2008), and an enhanced sense of personal worth (Klein, 2017). While these beneficial outcomes characterize providing support in general, recent studies have shown similar effects arising from specifically providing emotion regulation support to others (Zaki and Craig Williams, 2013; Nozaki, 2015; Reeck et al., 2016; Arbel et al., 2020; Cohen and Arbel, 2020; Nozaki and Mikolajczak, 2020; Arbel et al., 2022). These studies indicate both short- and long-term beneficial outcomes of EER for the provider’s emotional health (for review, see Cohen and Arbel, 2020). These beneficial outcomes among the general population include a reduction in depression symptoms and perseverative thinking (Morris et al., 2015), enhanced positive mood (Schacter and Margolin, 2019), and increased happiness (Morelli et al., 2015). For example, providing emotional support, such as empathy, to a same-sex friend improved the provider’s self-reported daily well-being, increased happiness, and decreased distress (Morelli et al., 2015). These effects carried over to the next day, albeit in a reduced form. Prisoners at a high-security prison reported a more positive mood one month after attempting to improve the feelings of others (Niven et al., 2012). Similarly, during the COVID-19 pandemic, psychological distress and daily negative mood of healthy young adults were reduced following a 3-week EER training that included providing support to others using cognitive reappraisal (Arbel et al., 2022). EER is also beneficial in reducing psychological distress during and following a stressful task (the Trier Social Stress Task; TSST; Kirschbaum et al., 1992, 1993). Providing EER by writing a supportive letter to a friend reduced physiological stress markers shortly afterwards (systolic blood pressure and alpha-amylase, but not cortisol or reported negative affect among healthy participants; Inagaki and Eisenberger, 2016). Although this research is still in its infancy, it suggests that EER may reduce physiological stress responses, especially sympathetic-related arousal (Inagaki and Eisenberger, 2016). A recent study that used text-based online communication has demonstrated that EER is most effective when people use emotion regulation strategies such as cognitive reappraisal and problem-solving, as well as when providing empathic responses (Nozaki and Mikolajczak, 2022).

Depression is characterized by impairments in social interactions (Teo et al., 2013), with better social relationships having a positive impact on mental health (Schön et al., 2009). The perception of support from others is commonly associated with a lower risk for depression and depressive symptoms (for review, see Gariépy et al., 2016). Throughout the life course, from childhood to old age, having a large social network is associated with a lower risk for depression (Santini et al., 2015; Loades et al., 2020). Individuals with depression may improve their social relationships by providing EER support to others. For example, providing support using cognitive reappraisal to individuals with high self-esteem who shared their failures was associated with increased positive affect, as well as higher relationship quality (Marigold et al., 2014). In emerging adulthood, EER was also found to improve relationship quality; a greater tendency to engage in taking perspective was associated with a higher quality of relationships and a greater well-being (Chan and Rawana, 2021). College students who favored EER strategies and perceived them to be useful were more well-adjusted, more connected socially, and developed stronger supportive relationships than their counterparts (Williams et al., 2018). Moreover, in new social contexts, EER can help a person become popular in both work and non-work interactions (Niven et al., 2015). Therefore, it is unsurprising that feelings of social connection increase when support is given (Inagaki and Ross, 2018).

Despite the evidence that EER may be a promising way to reduce depression symptoms, some findings suggest that under specific contexts EER may be maladaptive for depressed individuals. For example, providing tangible support to friends or listening to their disclosures led to higher levels of depression among young adults (Morelli et al., 2015). In addition, providing support to a stranger experiencing a negative event was found to be challenging and exhausting for providers (Gosnell and Gable, 2017). Furthermore, support providers who expressed high concerns about the effectiveness of their support displayed greater signs of depletion (Gosnell and Gable, 2017).

### 1.2. The mechanisms underlying the beneficial outcomes of EER in depression

Although providing support to others may be difficult or exhausting (Morelli et al., 2015; Gosnell and Gable, 2017), recent findings indicate that providing emotion regulation support to others is mostly beneficial for individuals with depression (see Table 1). These findings received support both in studies assessing providing EER to a stranger and in studies in which EER was provided to an acquaintance or a family member. For example, helping strangers by using cognitive reappraisal in a three-week training study among healthy young adults reduced the providers’ depression symptoms and perseverative thinking (Morris et al., 2015; Doré et al., 2017). These reductions were mediated by increased use of habitual reappraisal (see also Arbel et al., 2022).

**Table 1:** Summary of the existing empirical research on the benefits of EER to the provider.

Study	Population	Sample	Receiver	EER type	EER instructions	Outcome measures	Study design	Time frame	Main findings
Behavioral									
Arbel et al. (2022)	General	Exp 1: Other reappraisal group (*N* = 56, mean age = 27.02, 78% female) vs. control group. Exp 2: Other reappraisal group (mean age = 27.39, 59% female) vs. control groups	A stranger	Cognitive reappraisal	Help the person reframe the situation in order to feel better	daily negative mood (PANAS) and psychological distress (DASS)	online intervention	3 weeks	Reduction in psychological distress and daily negative mood
Doré et al. (2017)	General	*N* = 166 (mean age = 23.7 years, 71.7% female)	A stranger (on a web platform)	Cognitive reappraisal	(1) Offer empathy; (2) Identify cognitive distortions; or (3) Help users reframe negative situations in more positive and adaptive ways	Change in reappraisal (ERQ); Change in depression levels (CES-D); Change in perseverative thinking (PTQ)	Online intervention	3 weeks	Individuals who provided support more frequently demonstrated a reduction in depressive symptoms and preservative thinking. These effects were mediated by an increased use of reappraisal
Horn and Maercker (2016)	General	Couples; (Males; *N* = 7, mean age = 29.62 and Females *N* = 73, mean age 27.93)	Partner	Emotional support	extrinsic emotion regulation (everyday behavior in the relationship).	Adjustment disorder (ADNM) Depression - (CES-D), over the past few months on a daily basis.	Online study	30 min	Couples with a higher tendency to engage in co-reappraisal reported fewer depressive symptoms
Mongrain et al. (2018)	Subclinically depressed (mild to moderate)	*N* = 648 (mean age = 32.25 years, 67.1% female)	A close other (friend, relative, or significant other)	Act of kindness	Participants were asked to be helpful or loving by demonstrating kindness toward a close other	Depression CES-D; Satisfaction -SWLS	Ambulatory intervention	3 weeks	Kindness acts decreased depression among individuals with low agreeableness
Morelli et al. (2015)	General	*N* = 98 same-gender pairs (25 pairs of males, 24 pairs of females; mean age = 19.41)	Friends	Instrumental and emotional support	Instrumental helping behaviors and emotional support (empathy and responsiveness)	Loneliness (UCLA), perceived stress (PSS), anxiety, and happiness	Daily diary	2 weeks	Providing emotional support to a same-sex friend improved the provider’s self-reported daily well-being, increased happiness, and decreased distress. Results lasted the next day but to a lesser extent. Providing instrumental support was beneficial if the provider gave emotional support
Morris et al. (2015)	General	*N* = 166 (mean age = 23.7 years, 71.7% female)	A stranger (on a web platform)	Cognitive reappraisal	(1) Offer empathy; (2) Identify cognitive distortions; or (3) Help users reframe negative situations in ways that are more positive and adaptive	Change in reappraisal (ERQ); Change in depression levels (CES-D); Change in perseverative thinking (PTQ)	Online intervention	3 weeks	Individuals who provided reappraisal support more frequently showed a reduction in depression and preservative thinking, as well as an increase in habitual reappraisal.
Niven et al. (2012) (Study 1)	Prisoners and prison staff member	*N* = 61: 21 prison staff members (33.3% female) and 40 male prisoners (mean age = 38 years)	Inmates in prison	Inter-personal affect regulation	Affect improving strategies (complimenting, listening to problems, joking) and affect worsening strategies (criticizing, ignoring, using aggressive tones or words)	Affective well-being over the previous two weeks: enthusiasm depression and anxiety-contentment	Ambulatory intervention	1 month	Within one month, staff and prisoners who tried to improve others’ emotions reported improved well-being
Schacter and Margolin (2019)	General	*N* = 99 (Youth, mean age = 18.01, 45% female)	Friends and dating partners	Prosocial behaviors	Participants reported on their prosocial behaviors: standing up for someone, helping someone out, and making someone feel their thoughts and feelings are important	Positive and negative mood - PANAS	Daily diary	10 days	Participants who reported higher levels of depression expressed higher levels of positive mood on days when they were more prosocial
Physiological									
Inagaki and Eisenberger (2016)	Healthy	*N* = 51 (mean age = 21.02, 72.54% females)	Good friend	Supportive note	The participants were asked to give advice or comforting words	Heart rate, blood pressure, salivary alpha-amylase, salivary cortisol, self-reported stress, during the TSST and a following recovery phase	Laboratory study	During a stressor in the lab	Giving support reduced physiological stress in comparison to a control condition
fMRI									
Hallam et al. (2014)	Healthy	*N* = 20 (mean age = 23, 50% females)	A Stranger	Cognitive reappraisal, suppression	Providing emotional support to another person while watching a disturbing video	Brain activity, intensity of emotional experience	fMRI study	During an fMRI task	There was increased activation in fronto-parietal regions associated with emotion regulation (such as IFG) and empathy (such as mPFC and TPJ) following reappraisal
Inagaki and Eisenberger (2012)	Healthy	*N* = 20 females in long-term relationships	Male partner	Holding the partner’s hand as he receives an electrical shock (vs. control)	Supporting their partner by holding his hand	Brain activity, self-reported support effectiveness and social connectedness	fMRI study	During an fMRI task	Support was associated with increased activity in the ventral striatum and septal area; The septal area activity was negatively correlated with the activity of the amygdala

CESD, Centre for Epidemiological Studies Depression Scale; SWLS, Satisfaction With Life Scale; PANAS, Positive and Negative Affect Schedule; ERQ, Emotion Regulation Questionnaire; PTQ, Perseverative Thinking Questionnaire; UCLA, UCLA Loneliness Scale; PSS, Perceived Stress Scale; ADNM, Adjustment Disorder New Model; IER, Interpersonal Emotion Regulation questionnaire; TSST, Trier Social Stress Test.

Providing EER to someone close also reduced depression symptoms among the support providers. For example, high-security prisoners, who helped other inmates improve their mood, reported more positive emotions one month later (Niven et al., 2012). In addition, performing acts of kindness to someone close during a three-week intervention reduced depression among individuals with low agreeableness (Mongrain et al., 2018). Healthy women who experienced a recent major life stressor and engaged more in co-reappraisal with their partner (i.e., helping one another to regulate their emotions using cognitive reappraisal) showed lower depressive symptoms (Horn and Maercker, 2016). This effect was not observed among the male partners. This may possibly be due to women’s tendency to verbalize stress more frequently than men, suggesting that they rely more on EER than men to regulate their emotions. Additionally, depressed adolescents showed higher positive mood following days on which they provided support for dating partners or peers (Schacter and Margolin, 2019).

While these studies show the beneficial effects of EER in reducing depression, there are very few data on the mechanisms underlying these effects. Our comprehensive literature review suggests that EER enhances three key functions known to be impaired in depression: (1) cognitive empathy, (2) intrinsic emotion regulation, and (3) reward. Below, we review behavioral and neuroimaging evidence for the role of each of these mechanisms in EER and discuss how the recruitment of these processes during EER can lead to a reduction in symptoms of depression.

#### 1.2.1. Cognitive empathy

The first mechanism by which EER may reduce depression symptoms is empathy - the process of perceiving, understanding, experiencing, and responding to another’s emotional state (Barker, 2003). Researchers commonly distinguish between two types of empathy: cognitive empathy and emotional empathy (Davis, 1983). Cognitive empathy allows an individual to understand what someone else may think, adopt their psychological point of view, and predict their behavior (Frith and Singer, 2008). This ability may involve making inferences about the other’s affective and cognitive mental states (Shamay-Tsoory et al., 2009) and is related to cognitive flexibility (e.g., Eslinger, 1998; Shamay-Tsoory et al., 2004). Cognitive empathy, therefore, requires perspective-taking (Eslinger, 1998) and Theory of Mind (ToM; Shamay-Tsoory et al., 2004), which are the ability to understand another’s mental state and determine how the other will act (Decety and Jackson, 2004; Fan et al., 2011; Eres et al., 2015). Emotional empathy, on the other hand, enables individuals to tune into their feelings or experience affective reactions to the observed experiences of others (Shamay-Tsoory, 2011; Zaki, 2014). It is unconscious and requires lower-order cognitive abilities than cognitive empathy (Shamay-Tsoory, 2011; de Waal and Preston, 2017). High emotional empathy may be maladaptive due to the possibility of becoming overwhelmed by one’s distress and withdrawing from social interactions (Grynberg and Lopez-Perez, 2018). It has been suggested that the risk for depression and its severity may be associated with high emotional empathy levels and limited cognitive empathy (Schreiter et al., 2013).

Theoretical models suggest that EER requires the identification of the other person’s emotions (Reeck et al., 2016; Nozaki and Mikolajczak, 2020), making empathy a crucial antecedent of EER (Zaki, 2020). EER involves mostly cognitive empathy (Levy-Gigi and Shamay-Tsoory, 2017), as the individual needs to understand the other person’s perspective in order to choose which emotion regulation strategy can help that person (Gilead and Ochsner, 2021). Therefore, cognitive empathy is crucial during EER, as it enables the support provider to understand the other person’s perspective and to choose an emotion regulation strategy that fits the situation that the other is experiencing (Franklin-Gillette and Shamay-Tsoory, 2021). In line with this idea, in a study of dyadic interactions between romantic couples, the provider’s cognitive, but not emotional empathy, was linked to more successful EER for the support receiver (Levy-Gigi and Shamay-Tsoory, 2017). The importance of cognitive empathy in EER has also been highlighted in another study, which showed that cognitive empathy mediated the link between EER and higher couple satisfaction (Florean and Păsărelu, 2019). Together, these findings indicate the role of cognitive empathy in EER. Therefore, EER may be a good tool for elevating cognitive empathy among those characterized by deficits in cognitive empathy, such as individuals with depression. It is noteworthy, however, that the mentioned studies (Levy-Gigi and Shamay-Tsoory, 2017; Florean and Păsărelu, 2019) focused on the effects of support on the support receiver and not on the support provider. Therefore, more research is needed to understand the cognitive empathy’s role in EER in both healthy and clinical populations.

While the behavioral data on cognitive empathy in EER is limited, neuroimaging data support the role of cognitive empathy in EER. For example, Hallam et al. (2014) have found that providing EER increased activity in brain regions associated with empathy, such as the left tempero-parietal junction (TPJ), inferior temporal gyri, the medial prefrontal cortex (mPFC) and the temporal pole (TP). The mPFC and TPJ are likely to be engaged in the identification stage of EER (Reeck et al., 2016). Furthermore, the TPJ, mPFC, and TP are commonly associated with ToM (Gallagher and Frith, 2003; Saxe et al., 2006; Schurz et al., 2014). As EER is mainly associated with activations in the TPJ, mPFC and TP, it may depend more on cognitive empathy than on emotional empathy, which is mainly associated with activity in regions such as the insula, amygdala and anterior cingulate cortex (Dvash and Shamay-Tsoory, 2014).

Importantly, the cognitive empathy regions found activated during EER are considered key nodes of networks impaired in depression (Wen et al., 2021). For example, functional connectivity of the TPJ with the dorsolateral prefrontal cortex (dlPFC) and posterior cingulate cortex (PCC) is disrupted in depression (Penner et al., 2018). Patients with depression show abnormal large-scale functional coherence in the mPFC (Murrough et al., 2016). In addition, individuals with depression show less activity in the temporal pole than controls (Rodríguez-Cano et al., 2014), and this region appears to be reduced in size in this population (Rodríguez-Cano et al., 2014). Therefore, EER may be helpful for people with depression as it recruits the brain regions associated with cognitive empathy known to be impaired among these individuals (e.g., TPJ and mPFC; Shamay-Tsoory, 2011).

#### 1.2.2. Intrinsic emotion regulation

The second mechanism that may reduce depression symptoms following EER is the enhancement of intrinsic emotion regulation (IER) processes. Individuals with depression show IER deficits, tending to use maladaptive IER strategies (e.g., rumination, suppression) with a reduced tendency to use adaptive IER strategies (distraction, reappraisal) (Joormann and Stanton, 2016). These individuals are also considered inflexible regulators, i.e., they find it difficult to assess contextual demands, to fit the IER strategies they use to the situation at hand, and lack the ability to monitor the effectiveness of a chosen strategy and make necessary modifications (Chen and Bonanno, 2021). Accordingly, difficulty switching between IER strategies is associated with more severe symptoms of depression (Kato, 2015, 2017). Thus, EER may be particularly beneficial for individuals with depression as it can enhance their use of adaptive IER strategies.

In support of this idea, in a three-week training study, supporting others using cognitive reappraise increased the supporter’s tendency to employ reappraisal on their own experiences. This increase in the use of cognitive reappraisal was associated with a reduction in depression and perseverative thinking. Thus, beyond reducing depressive symptoms, helping others provides an opportunity to practice and improve one’s regulatory skills (Morris et al., 2015; Doré et al., 2017). This may also have an impact on the quality of social interactions. For example, people who were better at regulating emotions were found to have better social interactions and were more inclined to engage in prosocial behaviors (Lopes et al., 2005). Namely, people who struggle with social interactions might be able to interact with others more effectively and have more positive social interactions if they are trained in emotion regulation abilities. One reason that EER may be a good tool for practicing one’s regulatory skills, especially among individuals with depression, is based on the idea that EER may require less cognitive effort than self-regulation (Cohen and Arbel, 2020). Most adaptive ways people use to regulate their emotions require a relatively high amount of cognitive resources (Ortner et al., 2016), which are not always available during a depressive episode (Zetsche et al., 2012).

The idea that EER may be less demanding than self-regulation is supported by findings showing that speaking in a third-person language (e.g., “Why is Lisa feeling this way?”) leads to a greater reduction in negative affect than using a first-person language (e.g., “Why am I feeling this way?”). Third-person thinking enhances people’s ability to control their thoughts, feelings, and behavior during stressful situations (Kross et al., 2014; Moser et al., 2017; Nook et al., 2017; Streamer et al., 2017). Similarly, considering other people’s problems entails psychological distance, making EER easier than regulating one’s emotions. This psychological distance may also help navigate stressful experiences in more objective, wise, and emotionally intelligent ways (Beck, 1970; Mischel and Rodriguez, 1993; Fujita et al., 2006; Trope and Liberman, 2010; Kross and Ayduk, 2011; Grossmann and Kross, 2014; Bernstein et al., 2015). Importantly, speaking to oneself in a third-person language facilitates IER relatively effortlessly without draining the cognitive control resources that are depleted during stressful situations (Moser et al., 2017). A third account for the relative effectiveness of EER as compared to IER comes from Coan’s social baseline theory (SBT; Beckes and Coan, 2011). According to this theory, the human brain acts as if it is in a social environment, so being near other people or groups is our baseline. Based on this assumption, humans experience fewer negative outcomes when embedded in social groups than when excluded or alone (Beckes and Coan, 2011; Coan and Maresh, 2014). In support to the social nature of emotion regulation, an individual’s dorsolateral prefrontal cortex (dlPFC), which is important for the self-regulation of emotions, is less active when they are around supportive others. Namely, the brain has to spend less attentional resources on IER when around others (Beckes and Coan, 2011; Coan and Maresh, 2014; for a review, see Ochsner and Gross, 2008). Consequently, Beckes and Coan (2011) and Coan and Maresh (2014) argue that the use of EER is not only beneficial but also more effective and efficient than self-regulation since less cognitive resources are needed to employ the regulation. In line with this idea, Levy-Gigi and Shamay-Tsoory (2017) observed that EER is more effective than IER in reducing distress. Therefore, EER can serve as an effective means for increasing self-regulatory skills in depression (Liu, 2022).

Neuroimaging data, mainly from healthy individuals, support the idea that EER recruits brain networks involved in IER, which are impaired in depression. For example, when healthy participants are asked to regulate other peoples’ emotions watching a disturbing video using cognitive reappraisal or expressive suppression was associated with increased activity in brain regions involved in IER, such as the left anterior cingulate cortex (ACC) and the rostral and medial prefrontal cortex (Hallam et al., 2014). The ventrolateral prefrontal systems may be involved in the selection stage of EER, while the lateral prefrontal and posterior medial prefrontal regions may be involved in the implementation stage of EER (Reeck et al., 2016). Activity in the ACC, and the connectivity between it and other regions, such as the striatum and the insula, are impaired in depression (Philippi et al., 2015). Also, the volume of the gray matter of the subgenual ACC is significantly reduced in patients with depression (Drevets et al., 2008). In addition, patients who failed to recover during a 12-week controlled treatment had a smaller volume of gray matter in the ACC than remitted patients (Gunning et al., 2009), suggesting a better clinical outcome for patients with larger ACC (Frodl et al., 2008). The rostral and medial prefrontal cortex also show altered activity in depression (Rodríguez-Cano et al., 2014; Murrough et al., 2016).

In addition to ACC and prefrontal regions, EER is also associated with activation in the septal area (SA). Among healthy participants, SA activation is increased when emotional support is given to another person (holding the partner’s arm when he received an electrical shock; Inagaki and Eisenberger, 2012). The SA also appears to be involved in caregiving behaviors (D’Anna and Gammie, 2009), such as parental care (Inagaki, 2018) and IER (Singewald et al., 2011). Increased activation of this region while giving support is associated with greater perceived effectiveness of the support, increased social connectedness, and reduced amygdala activity (Inagaki, 2018). Reduction in amygdala activity is often seen in IER tasks (Walter et al., 2009) but less among depressed individuals (Johnstone et al., 2007), who are characterized by hyperactivation of the amygdala (Sheline et al., 2001; Surguladze et al., 2004; Suslow et al., 2010; Victor et al., 2010).

Hallam et al. (2014) and Inagaki and Eisenberger (2012) studied the brain mechanisms of EER in healthy individuals; we assume that the same IER networks are activated when individuals with depression provide EER. Thus, asking individuals with depression to provide emotion regulation support to others may strengthen brain regions associated with IER, whose activity is commonly impaired among this population.

#### 1.2.3. Reward

A third way in which EER may benefit individuals with depression is through the rewarding nature of this act. Reward represents the pleasure individuals typically experience when they receive or experience something positive (Wilson et al., 2018). Reduced reward function is a key diagnostic criterion for depression (Feighner et al., 1972). Individuals with depression show altered reward sensitivity (Pizzagalli et al., 2008). They are less responsive to social (e.g., positive feedback) and to non-social (e.g., monetary gains) rewards and are less motivated to seek rewards (Forbes et al., 2009, 2010; Pizzagalli et al., 2009; Olino et al., 2014, 2015). As a result, it may be difficult for a person with depression to avoid negative situations and to make new positive experiences to overcome a depressive mood. Individuals with depression may, therefore, especially benefit from rewarding experiences such as providing support to others (Morris et al., 2015; Horn and Maercker, 2016; Doré et al., 2017; Mongrain et al., 2018).

Extensive behavioral research has shown that prosocial behaviors enhance well-being (for reviews, see Anderson et al., 2014; Konrath, 2014; Aknin and Whillans, 2021). EER is mostly a prosocial act, suggesting that it can also serve as a rewarding experience (Aknin et al., 2018). And providing emotion regulation support to others does increase positive mood (Orth et al., 2016; Will et al., 2017) and boosts feelings of self-worth (Schacter and Margolin, 2019).

Neuroimaging data support the behavioral findings that EER is a rewarding experience for the support provider. Providing EER increases activation in the striatum (Inagaki and Eisenberger, 2012; Hallam et al., 2014), a region implicated in reward processing (Delgado, 2007). Helping other people using cognitive reappraisal is associated with heightened activity in the caudate nucleus (part of the dorsal striatum; Hallam et al., 2014). Similarly, activation increased in the ventral striatum (VS) when women held their partner’s hand while he was experiencing physical pain (electric shock; Inagaki and Eisenberger, 2012). The reward system is likely to be involved in the monitoring stage (Sheppes et al., 2015).

A growing body of literature suggests that the dopaminergic mesolimbic regions involved in reward processing during EER are dysfunctional in depression (Dunlop and Nemeroff, 2007). The striatum shows reduced activation in depression (Robinson et al., 2012), especially during reward processing (Kelley, 2004; O’Doherty et al., 2006; Delgado, 2007). Depression is also known to be associated with altered intrinsic connectivity within the ventral striatum (Pan et al., 2017). The striatum also appears smaller among patients with depression compared to controls (Dombrovski et al., 2012). Patients with depression who committed suicide showed decreased gray matter in the VS (Dombrovski et al., 2012). Taken together, these results highlight the potential role of VS recruitment during EER in reducing depressive symptoms.

## 2. Conclusion

Depression is a serious mental illness that adversely affects the person’s mood, behaviors and thoughts. This leads to personal and societal costs. Only 50% of people with depression benefit from psychological treatment and medications (Khan and Brown, 2015; Cuijpers et al., 2018). This review has shown that EER appears promising as a non-pharmaceutical treatment for depression. Behavioral and neuroimaging evidence both suggest that EER reduces depression symptoms and promotes well-being among the healthy population. These effects appear to be due to EER affecting three key processes that are impaired in depression: cognitive empathy, IER, and reward. Individuals with depression show altered neural activity in the brain regions subserving these cognitive functions as well as behavioral deficits. Initial findings indicate that the benefits of EER for depressed individuals are achieved by the recruitment of these three cognitive functions, but there is a need for more empirical research (Cohen and Arbel, 2020).

Other mechanisms, such as increased social relationships and connectedness, may also underlie the beneficial effects of EER for depressed individuals (Marigold et al., 2014). Individuals with depression may find it difficult to engage in social connections due to their own distress and anxiety, which may lead them to withdraw or avoid social interactions (Kupferberg et al., 2016). Asking these people to provide EER to another person may help them engage in social interactions, increase their feeling of social connectedness and prevent withdrawal or avoidance behaviors (Brown et al., 2012; Satici et al., 2016). Furthermore, when people with depression provide EER support to others, the feeling that they helped another person may enhance their self-efficacy (Caprara and Steca, 2007), known to be low in individuals with depression (Volz et al., 2019) and to be important in preventing depressive symptoms (Blazer, 2002). As there are only a few studies on the role of EER in increasing social connectedness and self-efficacy (Marigold et al., 2014), more research is needed to test whether these mechanisms play a role in the beneficial effects of EER for individuals with depression.

It is noteworthy, however, that people with depression may find it difficult to provide emotional support. Indeed, there is evidence showing that providing support may be exhausting for the provider (Gosnell and Gable, 2017). Supporting others is challenging and potentially stressful since it requires emotional resources and the ability to identify, prioritize, and respond appropriately to the other person’s needs (Jayamaha et al., 2021). Depression is characterized by the depletion of cognitive resources, which may lead to a feeling of inability to deal with stressful situations and poor coping strategies, especially in interpersonal situations (Hammen, 1991). Namely, people with depression symptoms may perceive stressful situations, especially in interpersonal contexts, as something they can cope with effectively (Nezu and Ronan, 1988; Herzberg et al., 1998; Caldwell et al., 2004; Keser et al., 2020). The need to cope in these situations may lead to distress and burnout that, in turn, reduce the quality of the support (Given et al., 2011; Bastawrous, 2013; Liu et al., 2020). However, although people with depression report lower use of adaptive IER strategies during both self-regulation and EER (Joormann and Stanton, 2016), they manage to implement these strategies effectively (similarly to non-depressed individuals) when instructed to do so in laboratory and training studies (Ellis et al., 2013; Smoski et al., 2014; Millgram et al., 2015; Platt et al., 2015). These findings suggest that while people with depression are less prone to use adaptive emotion regulation strategies, they can implement these strategies when instructed to do so (Liu and Thompson, 2017).

Our review raises important suggestions for future research and clinical practice. Group therapy may be highly effective for individuals with depression as this therapy can encourage both the sharing of negative feelings and supporting others with their struggles (McDermut et al., 2001; Truax, 2001; Kösters et al., 2006; Cuijpers et al., 2008), what may increase cognitive empathy and self-regulatory skills, as well as serve as a rewarding experience. Namely, helping others may allow depressed individuals to practice IER strategies and cognitive empathy skills and to experience reward from their involvement in a prosocial act. Utilizing EER in therapy may thus help to decrease distress and improve well-being. However, it is important to consider the fact that individuals with depression may have difficulty providing support, which may adversely affect the effectiveness of their support (Given et al., 2011; Bastawrous, 2013; Liu et al., 2020). This should be taken into account when considering the effects of EER provided by individuals with depression on the support recipient, such as when applying EER in clinical settings (e.g., group therapy).

To conclude, the current paper suggests that depressed individuals may benefit from EER *via* three processes: cognitive empathy, intrinsic emotion regulation, and reward. As most prior studies on EER were done on the general population and only one study tested the effects of EER among depressed individuals (Mongrain et al., 2018), more research is needed to uncover the mechanisms underlying the benefits of EER in depression.

## Author contributions

AM drafted the manuscript. NC provided critical revisions. All authors contributed to the article and approved the submitted version.

## Conflict of interest

The authors declare that the research was conducted in the absence of any commercial or financial relationships that could be construed as a potential conflict of interest.

## Publisher’s note

All claims expressed in this article are solely those of the authors and do not necessarily represent those of their affiliated organizations, or those of the publisher, the editors and the reviewers. Any product that may be evaluated in this article, or claim that may be made by its manufacturer, is not guaranteed or endorsed by the publisher.
